# Genome-wide changes in expression profile of murine endogenous retroviruses (MuERVs) in distant organs after burn injury

**DOI:** 10.1186/1471-2164-8-440

**Published:** 2007-11-28

**Authors:** Young-Kwan Lee, Alex Chew, Lauren Fitzsimon, Rita Thomas, David Greenhalgh, Kiho Cho

**Affiliations:** 1 Institute for Pediatric Regenerative Medicine, Shriners Hospitals for Children Northern California and Department of Surgery, University of California, Davis, Sacramento, CA 95817, USA

## Abstract

**Background:**

Previous studies have shown that burn-elicited stress signals alter expression of certain murine endogenous retroviruses (MuERVs) in distant organs of mice. These findings suggest that MuERVs may participate in a network of pathophysiologic events during post-burn systemic response. To gain a better understanding of the biological roles of MuERVs in post-burn systemic response, we examined the genome-wide changes in the MuERV expression profiles in distant organs and the biological properties of the putative-burn related MuERVs were characterized.

**Results:**

Female C57BL/6J mice were subjected to an approximately 18 % total body surface area flame burn and tissues (liver, lung, and kidney) were harvested at 3 hours and 24 hours after injury. The changes in the MuERV expression profiles in these tissues were examined by RT-PCR using a primer set flanking the non-ecotropic MuERV U3 promoter region within the 3' long terminal repeat. There were differential changes in the expression profiles of MuERV U3 regions after injury in all three tissues examined. Subsequently, a total of 31 unique U3 promoter sequences were identified from the tissues of both burn and no burn mice. An analysis of viral tropisms revealed that putative MuERVs harboring these U3 promoter sequences were presumed to be either xenotropic or polytropic. Some putative transcription regulatory elements were present predominantly in U3 promoter sequences isolated from burn and no burn mice, respectively. In addition, *in silico *mapping using these U3 sequences as a probe against the mouse genome database identified 59 putative MuERVs. The biological properties (coding potentials for retroviral polypeptides, primer binding sites, tropisms, branching ages, recombination events, and neighboring host genes) of each putative MuERV were characterized. In particular, 16 putative MuERVs identified in this study retained intact coding potentials for all three retroviral polypeptides (*gag, pol*, and *env*). None of the putative MuERVs identified in this study were mapped to the coding sequences of host genes.

**Conclusion:**

In this study, we identified and characterized putative MuERVs whose expression might be altered in response to burn-elicited systemic stress signals. Further investigation is needed to understand the role of these MuERVs in post-burn systemic pathogenesis, in particular, via characterization of their interaction with host genes, MuERV gene products, and viral activities.

## Background

Endogenous retroviruses (ERVs) are found in the genome of all vertebrates. They are derived from retroviral infections of germ-line cells followed by permanent incorporation, called colonization, into the host's genome. ERVs are transmitted vertically to the offspring as part of the parental genome by Mendelian order [1]. It is estimated that human ERVs (HERVs) and murine ERVs (MuERVs) constitute approximately 8 % and 10 % of their genomes, respectively [2,3]. The majority of ERVs have defective genomes as a result of the accumulation of deletional or insertional mutations as well as recombinations since their initial colonization. However, certain ERVs retain the full coding potentials for all or individual retroviral polypeptides [2,3]. It has also been well-documented that retroviral long terminal repeats (LTRs), which harbor unique U3 promoter and enhancer sequences, are capable of directly regulating the transcriptional activities (e.g., primary transcription, splicing, and polyadenylation) of neighboring host genes [4-6]. Therefore, in conjunction with the diversity of the ERV U3 promoter sequences, these findings infer active participation of ERVs in a range of normal physiologic as well as pathologic events of the host [7,8].

The association between ERVs and pathologic events underlying tumorigenesis and autoimmune diseases has been described in a number of reports [9,10]. For instance, expression of retroviral proteins from the human teratocarcinoma-derived virus (HTDV), a member of the HERV-K family, has been detected in tetratocarcinoma cell lines, breast cancer, and testicular tumors [11]. Further support for the ERVs' roles in various disease processes comes from studies that show some HERVs (HERV-H, HERV-W, and HERV-R) contain the intact envelope (*env*) gene capable of coding an *env *glycoprotein called syncytin [12-14]. Syncytin was originally identified as a fusogenic glycoprotein, which plays a crucial role in syncytiotrophoplast formation and placenta morphogenesis during periimplantation of embryos [15]. In recent studies, the proinflammatory properties of syncytin have been attributed to axonal demyelination, at least in part, during the development of autoimmune multiple sclerosis in humans [16]. In addition, HERVs are implicated in the pathogenesis of a number of other autoimmune diseases, such as schizophrenia, insulin-dependent diabetes mellitus, and systemic lupus erythematosus [17-19]. However, further investigations are needed to fully understand the roles of ERVs in these and other disease processes in humans and animals.

In our previous studies, we demonstrated that burn-elicited stress signals altered the expression of MuERVs in distant organs of mice in a tissue-specific manner [20-22]. These MuERVs had unique U3 promoter sequences suggesting different profiles of transcription regulatory elements in each of these sequences. Interestingly, some of these MuERVs are very similar in viral genome structure to the murine acquired immune deficiency syndrome (MAIDS) virus, which is known to cause immune disorders in infected mice [20,23]. These findings led to the hypothesis that burn-elicited stress signals are responsible, at least in part, for the genome-wide response of specific MuERVs. In addition, they may play causative roles in post-burn pathogenesis as well as in other stress-related disease processes. In this study, we identified putative MuERVs whose expression was altered in response to burn-elicited stress signals. Subsequently, the biological properties (coding potentials for retroviral polypeptides, primer binding sites (PBSs), viral tropism, branching ages, recombination events, and neighboring host genes) of these MuERVs were analyzed, and their roles in post-burn pathogenesis are discussed.

## Results

### Differential alterations in MuERV expression profiles in distant organs of mice after burn

**Figure 1:**
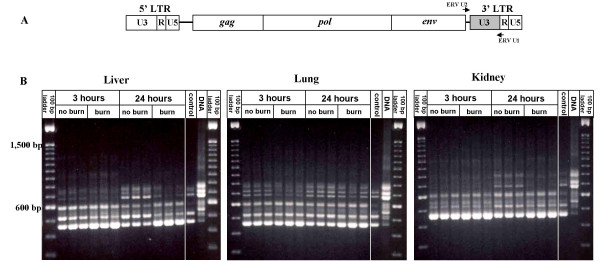
**Changes in mRNA expression profiles of MuERVs in distant organs after burn**. (A) Schematic representation of primer locations on MuERV. A set of primers (ERV-U2 and ERV-U1) flanking the 3' U3 region are indicated by arrows. (B) RT-PCR analysis of the MuERV expression after burn in the liver, lung, and kidney. Tissues (liver, lung, and kidney) harvested at 3 hours and 24 hours after 18 % TBSA burn were subjected to RT-PCR analysis of MuERV expression using a primer set flanking the 3' U3 region. Respective tissues harvested without any treatment, except for cervical dislocation, serve as a no treatment control in comparison to no burn controls (subjected to anesthesia, resuscitation, and CO_2 _inhalation). One representative sample out of three no treatment controls for each tissue is presented. In addition, a genomic MuERV profile was used as a reference.

To investigate the changes in genome-wide MuERV expression after burn, we examined the transcription profiles of non-ecotropic MuERV U3 regions in the liver, lungs, and kidney from both burn and no burn mice (female C57BL/6J) by RT-PCR (Figure 1). It needs to be noted that the no burn groups were subjected to anesthesia and fluid resuscitation. The U3 regions were selected for expression analysis because of their highly polymorphic sequences among the MuERV population compared to the rest of the viral genome. The U3 expression profiles of these individual tissues were compared to the genomic (C57BL/6J) profile as well as the expression profiles of corresponding tissues harvested without any treatment, such as anesthesia and resuscitation (no treatment control). Changes in the U3 expression profiles in all three tissues were evident at 24 hours after burn and these changes were tissue-specific. In addition, substantial differences in U3 expression profiles were observed in lung samples at 3 hours after burn (Figure 1B). These changes were manifested by either an increase or decrease in the expression of specific U3 regions after burn. The U3 regions amplified in this experiment, which include an approximately 120 bp of additional sequence upstream and downstream of the U3, were represented by seven major groups ranging in size from 461 bp to 798 bp. Interestingly, the U3 expression profiles of no burn controls (treated with anesthesia and resuscitation) of all three tissues at 3 hours were different at 24 hours. In addition, the U3 expression profiles of tissues without any treatment were substantially different from no burn controls at both time points. The difference in the U3 expression profiles among no burn controls at two different time points and no treatment controls suggests that anesthesia and resuscitation transiently affected the expression of MuERVs.

**Figure 2:**
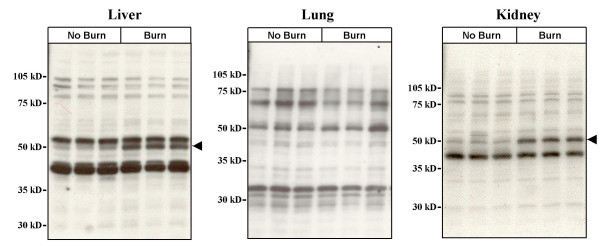
**Post-burn increases in expression of MuERV proteins reactive against MLV envelope antibody in distant organs**. Liver, lung, and kidney samples collected at 24 hours (burn and no burn) were analyzed for changes in MuERV protein expression by Western blot analysis using Rauscher MLV envelope antibody. Arrows indicate envelope antibody-reactive MuERV proteins in the liver and kidney whose expression is increased after burn.

To investigate whether there are post-burn changes in the expression of MuERV envelope protein, Western blot analyses were performed (Figure 2). In the liver, there was significantly increased reactivity in a band slightly smaller than 50 kD (arrow) at day 1 after burn. Similarly, induction of a reactive protein smaller than 50 kD was observed in the kidney at day 1. However, there were no significant changes in the lung. It is likely that the cleavage and/or truncation of the intact envelope protein products due to mutation may account for the presence of the apparently smaller than the expected size (~70 kD).

### Identification and characterization of differentially regulated MuERV U3 sequences after burn

**Figure 3:**
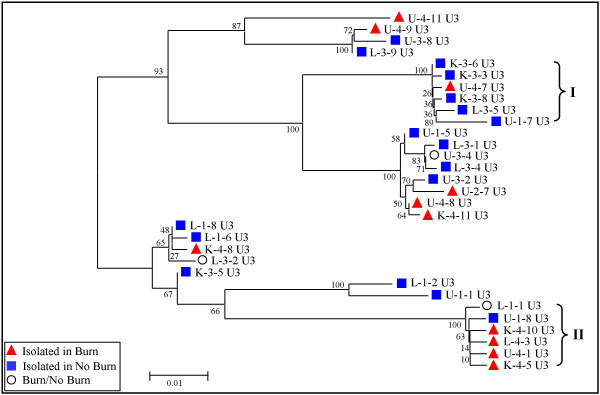
**Phylogenetic analysis of MuERV U3 sequences related to burn and/or no burn**. Based on the multiple alignment data in Additional file 1, Figure S1, the phylogenetic tree for MuERV U3 sequences was established using the neighbor-joining method. Branch lengths are proportional to the distance between the taxa, which are drawn to scale. The values at the branch nodes indicate the percentage support for a particular branching after 100 bootstrap replications were performed.

To determine genetic variations among differentially expressed MuERV U3 sequences in distant organs after a burn, the U3 sequences isolated from the RT-PCR products were subjected to multiple alignment and phylogenetic analyses. Amplified MuERV U3 sequences from each experimental group were subjected to cloning after purification of the PCR reactions. Clones from each group were picked for sequencing analysis primarily based on the differences in size. A total of 75 MuERV U3 sequences were initially cloned from all three tissues of both burn and no burn mice, and subsequent multiple alignment analyses identified 31 unique U3 sequences with nine different sizes (346 bp, 384 bp, 392 bp, 405 bp, 406 bp, 433 bp, 556 bp, 600 bp and 601 bp) (see Additional file 1, Figure S1). Of the 31 U3 sequences, 11 were isolated from tissues of burn mice, 17 were isolated from no burn mice, and three were from both burn and no burn mice. Both the 5'-end and 3'-end regions of the U3 sequences analyzed in this experiment were conserved, however, the sequences spanning the middle region were highly variable (see Additional file 1, Figure S1). The variations in this middle region included the presence and/or absence of several direct repeats and a 190 bp insertion. These variations may be directly associated with the different transcription potentials of each U3 promoter. It has been documented that MuERV tropism is closely linked to their U3 sequences. The tropism characteristics of each U3 sequence was determined by comparison analysis using the reference sequences (direct repeats, unique region, and 190 bp insertion) first reported by Tomonaga *et al*. [30]. A total of three intact direct repeats (1/1*, 5/5*, and 6/6*), one 190 bp insertion, and one unique sequence (2) were identified among the U3 sequences examined (see Additional file 1, Figure S1). In Additional file 1, Figure S1, two other direct repeats (3 and 4/4*), which were not identified or were partially identified in this experiment, were marked as a reference. Primarily, four direct repeats (1/1*, 4/4*, 5/5*, and 6/6*), one unique region (2), and one 190 bp insertion were used for the tropism analysis. No obvious difference was noted between groups of burn and no burn in regard to tropism. Table 1 summarizes the tropism analysis of all 31 U3 sequences, 14 polytropic and 17 xenotropic. The phylogenetic analysis of the U3 sequences yielded a significant branching pattern with bootstrap values of greater than 50. It revealed six major U3 groups, which paralleled the size of the U3 sequences (346 bp, 384 bp/392 bp, 406 bp, 433 bp, 556 bp, and 600 bp) (Figure 3). It was of interest to note that the 600 bp group (marked with "I") consists of predominantly U3 sequences isolated from no burn mice, in contrast to the 346 bp group (marked with "II"), which mostly were derived from burn mice. Comparison analysis of the sizes of differentially expressed U3 fragments (Figure 1) and U3 clones provided information regarding the origins of these U3 clones. Based on these comparison data, four U3 sequences isolated from burn mice (K-4-5 U3, K-4-10 U3, L-4-3 U3, and U-4-1 U3 within group II) were likely derived from the ~450 bp fragment (before trimming 120 bp of non-U3 sequences), which was induced 24 hours after burn (Figure 1). In addition, five U3 sequences in group I (K-3-3 U3, K-3-6 U3, K-3-8 U3, L-3-5 U3, and U-1-7 U3) were presumed to originate from the ~700 bp fragments (before trimming 120 bp non-U3 sequences) whose expression was evident in no burn control mice compared to burn mice (Figure 1). The L-1-1 U3 and U-1-8 U3 in the group II were likely to be derived from the baseline expression of the ~450 bp fragment expressed in no burn mice (3 hours). It is also possible that these U3 sequences could be associated with stress signals from anesthesia and resuscitation. Further comparison analysis of the RT-PCR data and U3 clones will elucidate the relations between each differentially expressed U3 fragment and U3 clones of different sizes examined in this study.

**Table 1:** Summary of tropism characteristics of 31 unique MuERV U3 sequences

**Group**	**U3**	**Direct repeat/Unique region**					**Tropism**
			
		1/1*	**2**	4/4*	5/5*	6/6*	
	**U-4-7 U3**	Poly	X-I, II, IV, Poly	Poly	Poly	X-I, Poly	**P-II**
	**U-4-8 U3**	Poly	X-III, Poly	Poly	Poly	X-I, II, III, IV, Poly	**P-I**
	**U-2-7 U3**	Poly	X-III, Poly	Poly	Poly	X-I, II, III, IV, Poly	**P-I**
	**K-4-11 U3**	Poly	X-III, Poly	Poly	Poly	X-I, II, III, IV, Poly	**P-I**
	**K-4-8 U3**	•	X-I, II, IV, Poly	X-II	X-II, III, IV	X-I, II, III, IV, Poly	**X-II**
**Isolated in Burn**	**U-4-9 U3**	•	X-I, II, IV, Poly	X-I	X-II, IIII, IV	X-I, Poly	**X-I**
	**U-4-11 U3**	•	X-I, II, IV, Poly	X-I	X-II, III, IV	X-I, II, III	**X-I**
	**U-4-1 U3**	•	X-I, II, IV, Poly	X-III	•	X-I, II, III, IV, Poly	**X-III**
	**K-4-10 U3**	•	X-I, II, IV, Poly	X-III	•	X-I, II, III, IV, Poly	**X-III**
	**K-4-5 U3**	•	X-I, II, IV, Poly	X-III	•	X-I, II, III, IV, Poly	**X-III**
	**L-4-3 U3**	•	X-I, II, IV, Poly	X-III	•	X-I, II, III, IV, Poly	**X-III**

	**U-3-4 U3**	Poly	X-III, Poly	Poly	Poly	X-I, II, III, IV, Poly	**P-II**
**Burn/No Burn**	**L-3-2 U3**	X-III	X-I, II, IV, Poly	X-II	X-II, III, IV	X-I, II, III, IV, Poly	**X-II**
	**L-1-1 U3**	•	X-I, II, IV, Poly	X-III	•	X-I, II, III, IV, Poly	**X-III**

	**U-1-7 U3**	X-II, III	X-I, II, IV, Poly	Poly	Poly	X-I, Poly	**P-II**
	**K-3-3 U3**	X-II, III	X-I, II, IV, Poly	Poly	Poly	X-I, Poly	**P-II**
	**K-3-6 U3**	X-II, III	X-I, II, IV, Poly	Poly	Poly	X-I, Poly	**P-II**
	**K-3-8 U3**	X-II, III	X-I, II, IV, Poly	Poly	Poly	X-I, Poly	**P-II**
	**L-3-5 U3**	X-II, III	X-II, III	Poly	Poly	X-I, Poly	**P-II**
	**L-3-1 U3**	Poly	X-III, Poly	Poly	Poly	X-I, II, III, IV, Poly	**P-I**
	**L-3-4 U3**	Poly	X-III, Poly	Poly	Poly	X-I, II, III, IV, Poly	**P-I**
**Isolated in No Burn**	**U-1-5 U3**	Poly	X-III, Poly	Poly	Poly	X-I, II, III, IV, Poly	**P-I**
	**U-3-2 U3**	Poly	X-III, Poly	Poly	Poly	X-I, II, III, IV, Poly	**P-I**
	**L-1-2 U3**	X-II, III	X-III, Poly	Poly	X-II, III, IV	X-I, II, III	**X-III**
	**U-1-1 U3**	X-II, III	X-III, Poly	X-III	X-II, III, IV	X-I, II, III	**X-III**
	**L-1-6 U3**	X-III	X-I, II, IV, Poly	X-III	X-II, III, IV	X-I, II, III, IV, Poly	**X-III**
	**L-1-8 U3**	X-III	X-I, II, IV, Poly	X-II	X-II, III, IV	X-I, II, III, IV, Poly	**X-II**
	**K-3-5 U3**	X-II, III	X-I, II, IV, Poly	X-II	X-II, III, IV	X-I, II, III	**X-II**
	**L-3-9 U3**	•	X-I, II, IV, Poly	X-I	X-II, III, IV	X-I, Poly	**X-I**
	**U-3-8 U3**	•	X-I, II, IV, Poly	X-I	X-II, III, IV	X-I, Poly	**X-I**
	**U-1-8 U3**	•	X-I, II, IV, Poly	X-III	•	X-I, II, III, IV, Poly	**X-III**

Dot indicates absence of a homology with reference sequences. Underline represents the reference type closest to individual U3 sequences examined. Naming scheme of U3 sequence: Letter (K-kidney; L-liver; U-lung), first number ("1"–3 hours control; "2"–3 hours burn; "3"–24 hours control; "4"–24 hours burn), and second number (clone number). *Direct repeat region.

### Comparative analysis of transcriptional potentials of 31 unique U3 promoter sequences

To gain insights into the transcription potential, the profile of putative transcription regulatory elements within each U3 promoter sequence was determined. A total of 73 putative transcription regulatory elements were identified within all 31 U3 promoter sequences using the database from Genomatix (Munich, Germany) (see Additional file 1, Table S1). Among these transcription regulatory elements, five (marked with "a") (HMGA1/2 [high mobility group A protein 1/2], Thing1/E47 heterodimer, C/EBPβ [CCAAT/enhancer binding protein β], PAX6 [paired-box-containing protein 6], and SZF1 [stem cell zinc finger protein 1]) were present predominantly in the U3 sequences isolated from burn mice. On the other hand, 15 elements (marked with "b"), such as NF-κB, c-Myb, gut-enriched Krueppel-like factor, and PPAR/RXR heterodimer, were mostly mapped to the U3 sequences originating from no burn mice. Further characterization of the specific roles of the transcription regulatory elements predominantly present in the U3 sequences from either burn or no burn mice in burn-elicited systemic pathogenesis is warranted.

### Genomic mapping and characterization of putative MuERVs harboring individual U3 sequences

In this experiment, the putative MuERVs harboring the individual U3 sequences within their LTRs were identified and mapped by a systemic search of the mouse genome database (C57BL/6J strain) using each U3 sequence as a probe. When the search homology was limited to ≥ 98%, different U3 probes often resulted in an overlapping set of putative MuERVs. A total of 59 unique putative MuERVs were identified and their genomic map was established (Table 2). The size (5'-end of 5' LTR to 3'-end of 3' LTR) of these MuERVs ranged between 5,312 bp and 9,054 bp. Among them, the U3 sequences of six putative MuERVs (marked with "c") (K-4-11.1b, U-4-8.1, U-4-8.5, U-4-8.11, U-4-8.18, and U-4-11.1) had 100 % homology with the respective U3 sequences isolated from burn mice. In addition, 13 putative MuERVs (marked with "b") harbored U3 sequences matching 100 % to their respective U3 sequences isolated from no burn mice. The precise location and orientation on the genome, proviral genome size, PBS, and coding potential for the three major retroviral polypeptides of each putative MuERV were also characterized. In regard to PBS, the 18 bp sequences located immediately downstream of 5' LTR were surveyed to determine the PBS of each putative MuERV. The tRNA^Gln ^(indicated as "Q" in the table) primer is known to be used by the reverse transcriptase of polytropic as well as modified polytropic MuERVs during replication. In contrast, the tRNA^Pro ^(indicated as "P" in the table) primer is predominantly used by ecotropic and xenotropic MuERVs. Except for the two putative MuERVs (L-3-9.5 and U-4-11.1) harboring a tRNA^Pro ^PBS, the rest (57 putative MuERVs) had a tRNA^Gln ^PBS. The results from the analyses of coding potentials revealed that the majority of putative MuERVs had a defective genome leading to a missing start codon and/or introduction of a premature stop codon. However, 16 of them retained intact open reading frames (ORFs) for all three retroviral polypeptides (*gag*, *pol*, and *env*), therefore, they were presumed to be full-length ERVs. Some of those defective MuERVs had intact ORFs for *gag*, *pol*, and/or *env *polypeptides. The putative MuERVs cloned by U3 probes, which were presumed to originate from the burn-induced U3 fragments, will be the primary focus for future studies.

**Table 2:** Genomic location, proviral size, primer binding site, and coding potential of putative MuERVs

**Virus**	**Contig number**	**Subsequence**	Chr*	**Orientation**	**Size (bp)**	PBS **	***gag***	***pol***	***env***
**^*a *^L-1-1.4**	NT_039267.6	2466483-2472149	4	+	5667	Q	*P*	*P*	*-*
**^*a *^L-1-1.8**	NT_078575.5	18237577-18243244	8	+	5668	Q	*P*	*P*	*-*
**L-1-2.8a**	NT_039460.6	4167673-4158940	8	-	8734	Q	*+*	*+*	*+*
**L-1-2.8b**	NT_078575.5	12483528-12477141	8	-	6388	Q	*+*	*P*	*P*
**L-1-2.9**	NT_039472.6	28499787-28493004	9	-	6784	Q	*-*	*+*	*+*
**L-1-2.14**	NT_039606.6	27905917-27913739	14	+	7823	Q	*P*	*+*	*+*
**L-1-2.19**	NT_039687.6	54108037-54099311	19	-	8727	Q	*+*	*+*	*+*
**^*a *^L-3-2.4**	NT_039267.6	3529917-3535228	4	+	5312	Q	*+*	*P*	*P*
**^*b *^L-3-9.5**	NT_165760.1	8102397-8093731	5	-	8667	P	*+*	*+*	*+*
**^*b *^U-1-5.5a**	NT_039305.6	7470573-7479554	5	+	8982	Q	*P*	*+*	*+*
**^*b *^U-1-5.5b**	NT_109320.3	817587-826568	5	+	8982	Q	*+*	*+*	*+*
**^*b *^U-1-5.15**	NT_039621.6	37681733-37674548	15	-	7186	Q	*P*	*P*	*+*
**^*b *^U-1-5.16**	NT_039625.6	10813766-10804785	16	-	8982	Q	*+*	*P*	*P*
**^*b *^U-1-5.X**	NT_039700.6	8642080-8651060	X	+	8981	Q	*+*	*P*	*+*
**^*b *^U-1-7.1**	NT_078297.5	46243984-46253037	1	+	9054	Q	*P*	*+*	*+*
**^*a *^U-3-4.8**	NT_078575.5	47244198-47236723	8	-	7476	Q	*P*	*P*	*+*
**K-3-3.2**	NT_039202.6	12947290-12938249	2	-	9042	Q	*P*	*+*	*+*
**K-3-3.3a**	NT_039240.6	16281352-16289068	3	+	7717	Q	*+*	*P*	*P*
**K-3-3.3b**	NT_039240.6	101357871-101366913	3	+	9043	Q	*+*	*+*	*+*
**K-3-3.4**	NT_109315.3	1334988-1327627	4	-	7362	Q	*P*	*P*	*-*
**K-3-3.5a**	NT_078458.5	8656480-8663841	5	+	7362	Q	*P*	*P*	*-*
**K-3-3.5b**	NT_039305.6	6544154-6553194	5	+	9039	Q	*+*	*+*	*+*
**K-3-3.5c**	NT_109320.3	32960612-32953147	5	-	7466	Q	*P*	*P*	*-*
**K-3-3.10a**	NT_039490.6	1628826-1619788	10	-	9039	Q	*P*	*P*	*P*
**K-3-3.10b**	NT_039492.6	15143818-15152862	10	+	9045	Q	*+*	*+*	*+*
**K-3-3.11a**	NT_096135.4	25877081-25869722	11	-	7360	Q	*P*	*P*	*-*
**K-3-3.11b**	NT_096135.4	41839881-41848925	11	+	9045	Q	*+*	*+*	*+*
**K-3-3.11c**	NT_096135.4	52173389-52182429	11	+	9041	Q	*+*	*+*	*+*
**K-3-3.11d**	NT_165773.1	14484140-14493181	11	+	9042	Q	*P*	*P*	*+*
**K-3-3.13**	NT_039578.6	10683833-10691619	13	+	7787	Q	*+*	*+*	*P*
**K-3-5.13**	NT_039589.6	14019544-14010858	13	-	8687	Q	*+*	*+*	*+*
**^*b *^K-3-6.4**	NT_039258.6	12169957-12163352	4	-	6606	Q	*P*	*P*	*P*
**^*b *^K-3-6.5**	NT_165760.1	9622092-9631063	5	+	8972	Q	*+*	*P*	*P*
**^*b *^K-3-6.6**	NT_039343.6	25557034-25549676	6	-	7359	Q	*+*	*P*	*P*
**^*b *^K-3-6.7**	NT_039428.6	4390808-4399848	7	+	9041	Q	*+*	*+*	*+*
**^*b *^K-3-6.8**	NT_078575.5	50511987-50519348	8	+	7362	Q	*+*	*P*	*P*
**^*b *^K-3-6.12**	NT_039551.6	28009046-28018086	12	+	9041	Q	*P*	*+*	*+*
**K-4-11.1a**	NT_039185.6	8055063-8060808	1	+	5746	Q	*+*	*P*	*-*
**^*c *^K-4-11.1b**	NT_039189.6	8417864-8426844	1	+	8981	Q	*+*	*P*	*P*
**K-4-11.2**	NT_039206.6	34645399-34636419	2	-	8981	Q	*+*	*+*	*+*
**K-4-11.4a**	NT_039264.5	1995942-1986961	4	-	8982	Q	*+*	*P*	*+*
**K-4-11.4b**	NT_039264.5	8278820-8269838	4	-	8981	Q	*+*	*+*	*+*
**K-4-11.7a**	NT_039385.6	3682978-3673997	7	-	8982	Q	*P*	*P*	*-*
**K-4-11.7b**	NT_039413.6	9420528-9429226	7	+	8699	Q	*+*	*+*	*P*
**K-4-11.7c**	NT_039413.6	10493961-10502941	7	+	8981	Q	*+*	*P*	*-*
**K-4-11.7d**	NT_039433.6	34095286-34100959	7	+	5764	Q	*P*	*P*	*P*
**K-4-11.10a**	NT_039492.6	961582-954449	10	-	7134	Q	*P*	*P*	*P*
**K-4-11.10b**	NT_039492.6	33845187-33854167	10	+	8981	Q	*+*	*+*	*+*
**K-4-11.11a**	NT_039515.6	5820301-5829280	11	+	8980	Q	*+*	*+*	*+*
**K-4-11.11b**	NT_165773.1	297443-290657	11	-	6787	Q	*+*	*P*	*+*
**K-4-11.12**	NT_039548.6	17945382-17951142	12	+	5761	Q	*P*	*P*	*-*
**K-4-11.13**	NT_039590.6	6969427-6960447	13	-	8981	Q	*+*	*P*	*+*
**K-4-11.19**	NT_039687.6	31559507-31566568	19	+	7062	Q	*P*	*P*	*-*
**K-4-11.X**	NT_039706.6	194264-203244	X	+	8981	Q	*+*	*+*	*+*
**^*c *^U-4-8.1**	NT_039185.6	26088376-26097356	1	+	8981	Q	*+*	*P*	*+*
**^*c *^U-4-8.5**	NT_039324.6	798268-807248	5	+	8981	Q	*P*	*+*	*+*
**^*c *^U-4-8.11**	NT_039515.6	3658394-3649425	11	-	8970	Q	*+*	*P*	*+*
**^*c *^U-4-8.18**	NT_039678.6	472572-481451	18	+	8880	Q	*P*	*+*	*+*
**^*c *^U-4-11.1**	NT_039185.6	14772777-14781433	1	+	8657	P	*+*	*P*	*+*

Putative MuERVs isolated with 100% homology with respective U3 probes: ^*a*^Isolated in Burn/No Burn, ^*b*^Isolated in No Burn, and ^*c*^Isolated in Burn. The last number of putative MuERVs derived from the same U3 sequences indicates different chromosomal locations, and the letter next to this number represents different virus isolates. The coding potentials for retroviral polypeptides are indicated by ("+" intact/full-length), ("-" defective), and ("P" partial). * chromosome number, **primer binding site, P (tRNA^proline^), and Q (tRNA^glutamine^).

### Examination of evolutionary relationship among putative MuERVs by phylogenetic analysis of their reverse transcriptases

**Figure 4:**
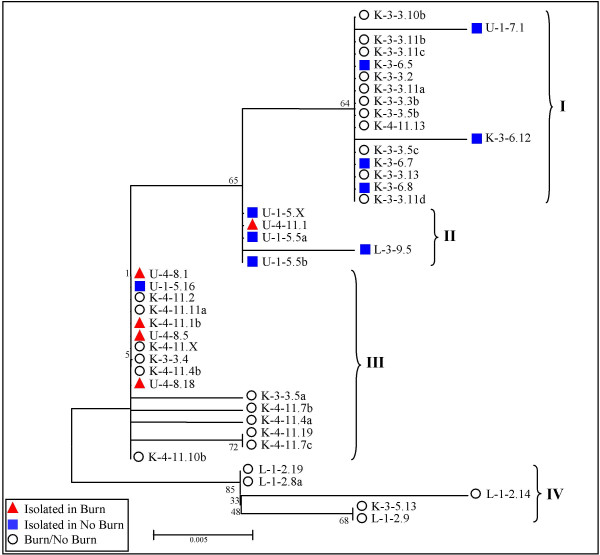
**Phylogenetic relationship of RTs of putative MuERVs**. Seven conserved domains of full-length RT sequences from 42 putative MuERVs were subjected to a phylogenetic analysis. The phylogenetic tree was established using neighbor-joining methods. The branch length represents a degree of divergence between RT sequences. The confidence values at the branch nodes indicate the percentage support for a particular branching following 100 bootstrap replications.

Due to the highly conserved nature of the reverse transcriptase (RT) among different retroviruses compared to the rest of viral proteins, the RT sequence has been used to determine phylogenetic relationships among retroviruses as well as other retroelements [24,25]. To examine the evolutionary relationship of the putative MuERVs identified in this study, a phylogenetic tree of RT sequences was constructed following a multiple alignment analysis (Figure 4). This phylogenetic analysis was based on the seven conserved domains of the RT, a total of 178 amino acids, which served as a reference for a number of evolutionary studies [24]. Among the 59 putative MuERVs, only 42 of them were subjected to the analysis because the other 17 MuERVs had an incomplete RT due to a deletion and/or premature stop. It appeared that they were subgrouped into four main branches (I ~ IV). The putative MuERVs derived from the same U3 probes tended to cluster into the same branches. Interestingly, four out of the five putative MuERVs (red triangle) identified using the U3 probes from burn mice (K-4-11, U-4-8, and U-4-11) clustered into a unique branch (III), implicating a close evolutionary relationship. It needs to be noted that these MuERVs had 100 % sequence homology with the respective U3 probes used for mapping.

### Examination of tropism of 16 putative full-length MuERVs by restriction fragment length polymorphism analysis

**Figure 5:**
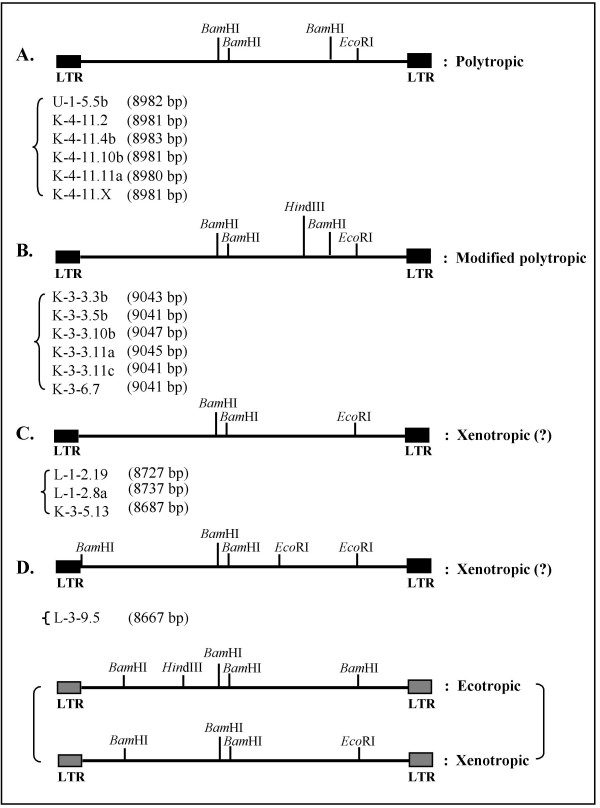
**Tropism analysis of putative full-length MuERVs**. Cellular tropism of 16 putative full-length MuERVs was determined by *in silico *RFLP analysis using three restriction enzymes (*Bam*HI, *Eco*RI, and *Hin*dIII). Relative locations of each restriction enzyme site were mapped on each MuERV. Expected RFLP patterns of ecotropic and xenotropic MuERVs are presented on the bottom as a reference [27].

The MuERV coding sequences are relatively conserved except for the *env *sequences. For instance, the *env *amino acid sequences of ecotropic MuERVs are substantially divergent from the other classes (xenotropic, polytropic, and modified polytropic) [26,27]. MuERVs can be grouped into four different classes (ecotropic, xenotropic, polytropic, and modified polytropic) based on restriction fragment length polymorphism (RFLP) following digestion with three enzymes (*Bam*HI, *Eco*RI, and *Hin*dIII) [27]. In this experiment, 16 putative full-length MuERVs with intact coding potentials for all three polypeptides were subjected to an *in silico *RFLP analysis to determine their tropism (Figure 5). Six (U-1-5.5b, K-4-11.2, K-4-11.4b, K-4-11.10b, K-4-11.11a, and K-4-11.X) of them were polytropic and another six (K3-3.3b, K-3-3.5b, K-3-3.10b, K-3-3.11a, K-3-3.11c, and K-3-6.7) were modified polytropic. The RFLP tropism data (polytropic and modified polytropic) was consistent with the results regarding the tropism of the corresponding U3 probes/sequences (Table 1 and Figure 5). In addition, four putative MuERVs (L-1-2.19, L-1-2.8a, K-3-5.13, and L-3-9.5) had unique RFLP profiles, which did not match with the reference, suggesting "xenotropic-like" tropism [26,27]. Further investigation is necessary to confirm the tropism of these four "xenotropic-like" putative MuERVs.

### Genetic evidence of recombination events in certain putative MuERVs

There are two unique features of MuERVs: two identical (at least at the time of integration) copies of flanking LTRs and a short direct repeat created at the genomic target during integration. They serve as indicators of integration ages as well as genomic recombination events that occurred in the host after the initial integration. The integration age of MuERVs can be estimated based on the degree of accumulated mutations within the presumed to be identical flanking LTRs of each MuERV. Among the 59 putative MuERVs examined, only 6 of them had mutations within each pair of LTRs ranging from 0.1349 % to 0.2869 % (Table 3). The integration ages of these MuERVs were calculated based on a formula of "0.13 % mutation rate between two flanking LTRs/one million years (MYr)" [28]. The integration ages of the putative MuERVs analyzed in this study ranged from 1.037 MYr to 2.206 MYr. In addition, as an indicator of genomic rearrangements between MuERVs as well as in other parts of the genome, we examined a short stretch of sequences (4 bp to 12 bp) flanking each MuERV for a direct repeat. It turned out that 11 out of 59 MuERVs were not flanked by a direct repeat, indicating that they were formed via recombination between two different MuERVs (Table 3). The rest had direct repeats of 4 bp except for the U-1-5.15 MuERV, which had direct repeats of 12 bp.

**Table 3:** Integration age and recombination event of putative MuERVs.

**Virus**	**LTR (bp)**	**Mutation rate (%)**	Integration age (MYr*)	**Direct repeat**	**Recombination**	**Virus**	**LTR (bp)**	**Mutation rate (%)**	Integration age (MYr*)	**Direct repeat**	**Recombination**
**L-1-1.4**	487	0	•	CCTT	no	**K-3-5.13**	547	0	•	GTAC	no
**L-1-1.8**	487	0	•	ATAT	no	**K-3-6.4**	741	0.1349	1.037	CAGG	no
**L-1-2.8a**	573	ND	ND	GGTC/GTCT	yes	**K-3-6.5**	741	0	•	ATAC	no
**L-1-2.8b**	574	0	•	GTAT	no	**K-3-6.6**	741	ND	ND	ACAA/ACAC	yes
**L-1-2.9**	574	ND	ND	GGAA/GGGG	yes	**K-3-6.7**	741	0	•	CCTG	no
**L-1-2.14**	574	0.1742	1.34	GTAT	no	**K-3-6.8**	741	ND	ND	GGAA/GGTG	yes
**L-1-2.19**	574	0	•	CTTG	no	**K-3-6.12**	741	0	•	AGAC	no
**L-3-2.4**	547	0	•	AACA	no	**K-4-11.1a**	698	0	•	CAAG	no
**L-3-9.5**	533	0	•	CTGG	no	**K-4-11.1b**	697	0	•	GTTG	no
**U-1-5.5a**	697	0	•	CCAC	no	**K-4-11.2**	697	0	•	GTGT	no
**U-1-5.5b**	697	0	•	CACC	no	**K-4-11.4a**	697	0.1434	1.103	CACC	no
**U-1-5.15**	697	0	•	AAACAAACAAAC	no	**K-4-11.4b**	698	0.1432	1.101	AAAC	no
**U-1-5.16**	697	0	•	CTGG	no	**K-4-11.7a**	697	0.2869	2.206	ATGA	no
**U-1-5.X**	697	0	•	ATAC	no	**K-4-11.7b**	697	0	•	ATTT	no
**U-1-7.1**	748	0.2673	2.056	ACAC	no	**K-4-11.7c**	697	0	•	CATG	no
**U-3-4.8**	697	0	•	AGGT	no	**K-4-11.7d**	698	0	•	CTAA	no
**K-3-3.2**	741	0	•	ATTG	no	**K-4-11.10a**	697	0	•	GTGC	no
**K-3-3.3a**	742	0	•	ACTT	no	**K-4-11.10b**	697	0	•	GATG	no
**K-3-3.3b**	742	0	•	ATGT	no	**K-4-11.11a**	697	0	•	ATAG	no
**K-3-3.4**	741	ND	ND	CCAA/CCTG	yes	**K-4-11.11b**	697	0	•	GGAG	no
**K-3-3.5a**	741	0	•	GATG	no	**K-4-11.12**	697	0	•	CTGT	no
**K-3-3.5b**	741	ND	ND	ATAT/TTAT	yes	**K-4-11.13**	697	0	•	GTGG	no
**K-3-3.5c**	741	0	•	ATAG	no	**K-4-11.19**	697	0	•	CTTC	no
**K-3-3.10a**	741	ND	ND	ACAG/TTAG	yes	**K-4-11.X**	697	ND	ND	TGAA/GAGT	yes
**K-3-3.10b**	743	ND	ND	CTGC/TTGC	yes	**U-4-8.1**	697	0	•	TTTG	no
**K-3-3.11a**	741	0	•	ACAC	no	**U-4-8.5**	697	0	•	AGGG	no
**K-3-3.11b**	743	ND	ND	AGGG/TTGG	yes	**U-4-8.11**	697	0	•	GTTC	no
**K-3-3.11c**	741	0	•	ACAC	no	**U-4-8.18**	697	0	•	CCTG	no
**K-3-3.11d**	741	ND	ND	AAAC/GAAA	yes	**U-4-11.1**	525	0	•	AACC	no
**K-3-3.13**	741	0	•	CTAC	no						

Dots indicate insufficient data to calculate integration ages. Absence of direct repeats at both ends of each MuERV indicates a recombination event. Integration ages were calculated based on a formula (0.13% mutation rate between two flanking LTRs = 1 MYr). ND (not determined); * Million years

### Host genes near the integration sites of putative MuERVs

The U3 promoter and enhancer sequences residing in the MuERV LTRs often regulate the transcriptional activities of neighboring host genes at different levels (e.g., primary transcription, splicing, and polyadenylation) [4-6]. To gain insights regarding potential roles of the putative MuERVs identified in this study, host genes within 110 kb upstream and downstream from the integration sites of each putative MuERVs were identified (Table 4). Nine putative MuERVs were integrated within the introns of various types of host genes, either in the same or opposite orientation. This suggests that expression of these host genes might be controlled by the U3 promoter and enhancer sequences of the respective putative MuERVs. In addition, at least one known host gene was identified from the 42 putative MuERVs (total of 145 genes) within the search range (see Additional file 1, Table S2). For instance, the K-3-3.13 putative MuERV was integrated near a cluster of various isotypes of histone genes on chromosome 13. Other host genes found near the integration sites of selective putative MuERVs include Selp, F5, Fcgr3a, and Scpep1, which are known to participate in various physiologic processes, such as inflammation and vascular wall homeostasis [31-34]. Although these putative MuERVs are not located within the host genes, their LTRs are still capable of controlling the transcriptional activities of nearby host genes. No known host genes were mapped near the integration sites of the last eight putative MuERVs within the search range.

**Table 4:** Location and orientation of putative MuERVs integrated into host genes.

**Virus**	Chr*	**Gene**	**Description**	**Genomic location**		**Insertion/Exons**	**Orientation**
						
				**virus (bp)**	**gene (bp)**		**virus/gene**
**U-1-7.1**	1	Ctse	Cathepsin E	133470113-133479149	133465860-133503051	Intron 1-2/10 exons	+/+
**K-3-3.3a**	3	Rsrc1	Arginine/serine-rich coiled-coil 1	67184007-67191723	67073648-67446326	Intron 4-5/10 exons	+/+
**L-3-2.4**	4	Ccdc21	Coiled-coil domain containig 21	133431467-133436778	133403174-133459161	Intron 3–4/14 exons	+/-
**K-3-6.5**	4	Galnt11	Polypeptide N-acetylgactosaminyltransferase11	24740764-24749735	24732958-24775983	Intron 1–2/12 exons	+/+
**L-1-1.8**	8	Chd9	Chromodomain helicase DNA binding protein 9	93776396-93782063	93718942-93944613	Intron 2–3/40 exons	+/+
**K-3-3.11b**	11	Abr	Active BCR-related gene	76365003-76374032	76232929-76438420	Intron 1–2/23 exons	+/-
**K-3-3.11d**	11	Plcd3	Phospholipase C, delta 3	102899753-102908794	102886394-102917748	Intron 1–2/16 exons	+/-
**K-4-11.11a**	11	Pkd1l1	Polycystic kidney disease 1 like 1	8820301-8829280	8726711-8873269	Intron 16–17/50 exons	+/-
**L-1-2.19**	19	Gprk5	G protein-coupled receptor kinase 5	60979970-60988696	60945139-61147005	Intron 1–2/16 exons	-/+

* chromosome.

## Discussion

Burn-elicited stress signals are directly linked to pathologic changes in distant organs contributing to systemic inflammatory responses syndrome and often multiple organ failure [29,30]. The results from our previous studies demonstrated that the expression of certain MuERVs was differentially altered in various tissues of mice after burn, suggesting that MuERVs may play a role in post-burn pathologic changes. Involvement of ERVs in inflammatory disease processes was exemplified by the direct role of syncytin, an envelope protein of HERV-W, in the development of multiple sclerosis, an autoimmune disease [16]. In addition, ERVs are implicated in an array of other diseases such as breast cancer, schizophrenia, rheumatoid arthritis, IDDM, myeloid leukemia, and T cell lymphoma [9,17-19,31-34]. However, a substantial amount of further studies are essential to gain clear insights into the ERVs' role in a number of disease processes, including systemic response after burn injury.

In this study, we confirmed that burn-elicited stress signals alter the expression of certain MuERVs in a U3 promoter- and tissue- specific manner. During the course of this study, 31 unique MuERV U3 sequences were identified from distant organs (liver, ling, and kidney) of burn and no burn mice. Size-based comparison analysis of the differentially expressed U3 fragments (RT-PCR products) and 31 U3 clones allowed us to determine whether the U3 clones are derived from the burn-induced U3 fragments. Each U3 promoter sequence had a unique transcription potential. It is likely that burn-elicited stress signals alter the intracellular transcription environment by activation or inactivation of certain transcription factors in a cell type- and tissue-specific manner, thereby, leading to differential genome-wide regulation of specific MuERVs. The MuERVs that were induced after a burn may have a pathophysiologic role in the systemic response different from repressed MuERVs. It was of interest to note that the U3 expression profiles in all three tissues of no burn mice (subjected to anesthesia and resuscitation only) at 3 hours were significantly different from the profiles at 24 hours. In addition, the U3 profiles of no burn mice at both time points did not match corresponding control tissues harvested without any treatment. These findings suggest that the initial treatments (anesthesia and resuscitation) during the burn procedure contributed to changes in the U3 expression profiles in a tissue-specific manner. Furthermore, the genomic U3 profile was distantly related to the U3 expression profiles from both burn and no burn mice, including mice without any treatment. It suggests that not all MuERVs on the mouse genome were actively transcribed and/or responded to stress signals in the tissues examined in this study. It will be worthwhile to perform a comprehensive examination of the MuERV expression profile in various cell types as well as tissue types.

Several transcription regulatory elements were identified more frequently in U3 sequences isolated from burn mice. These include binding sites for HMGA1/2, C/EBPβ, PAX6, SZF1, and Thing1/E47 heterodimer. These genes are known to participate in various normal as well as pathologic processes, such as SZF1's role in hematopoiesis and the involvement of C/EBPβ in the regulation of proinflammatory genes [35-37]. In addition, recent reports demonstrated that HMGA1 functions as a mediator of the development of sepsis, as evidenced by its increased serum levels in patients with septic shock [38]. Phylogenetic analysis revealed that four of the U3 sequences (K-4-5 U3, K-4-10 U3, L-4-3 U3, and U-4-1 U3) whose transcriptional activities are presumed to be induced in burn mice were clustered into a unique branch (Figure 3) and all have the HMGA1/2 binding element. We can speculate that the elevated systemic levels of HMGA1 protein during burn-elicited septic development may enhance transcriptional activities of certain MuERV U3 promoters, such as K-4-5 U3, K-4-10 U3, L-4-3 U3, and U-4-1 U3, through the HMGA1/1 binding element. It will be necessary to determine whether the transcription regulatory elements, predominantly present on the U3 promoters from burn mice, interact with corresponding proteins which in turn result in altered transcriptional activities. In addition, two hormone-related binding elements for steroidogenic factor 1 and estrogen-related receptor 1, were identified in both burn and no burn groups. It is possible that these elements may play a role in MuERV responses to changes in hormone levels due to various types of stress signals [39-41]. Since there is an increase in systemic glucocorticoid levels after burn, we tried to find a glucocorticoid response element on the U3 promoters isolated from burn mice, but we were not able to.

Almost all ERVs are considered to be defective in their genomic organization, and as a result, they are not capable of encoding intact retroviral polypeptides and are replication-incompetent. In this study, *in silico *mapping/cloning experiments using the 31 U3 sequences as a probe revealed 59 putative MuERVs. Among them, 16 putative MuERVs retained coding potentials for all three retroviral polypeptides and at least 12 of them were classified as polytropic or modified polytropic in regard to their tropism. These findings indicate that a substantial fraction of the MuERV population on the mouse genome is capable of encoding functional proteins and can infect host cells when they are activated for replication. Another group of putative MuERVs, defective in their genome structures, had intact coding potentials for *gag*, *pol*, and/or *env *polypeptides and it is likely that changes in the expression of these individual proteins affect the host cells' normal physiology, such as overexpression of syncytin in the brain of multiple sclerosis patients. It might be necessary to further characterize the biological roles of MuERVs presumed to be derived from U3 probes induced after burn.

One of the key findings from this study was that the majority of putative MuERVs are integrated into introns or near the host genes, suggesting that burn-mediated regulation of some of these MuERVs may be linked to the expression of neighboring host genes. The U-1-7.1 putative MuERV was integrated between exon 1 and exon 2 of the cathepsin E gene, which is essential for immune defense against microbial pathogens via its protease activity [42]. Interestingly, the U-1-7 U3 probe was derived from the lung and cathepsin E was differentially expressed in the lung compared to other tissues, such as kidney [43]. In addition, the L-1-1.8 putative MuERV derived from the L-1-1 U3 probe (isolated from the liver of no burn mice) was integrated between exon 2 and 3 of chromosome domain-helicase-DNA-binding protein 9 (CHD9), a chromosome remodelling factor [44,45]. Our recent study provided evidence suggesting a potential chromosomal remodelling in the liver after burn [46]. It will be of interest to investigate whether the U3 promoter and enhancer sequences of the L-1-1.8 putative MuERV affect the expression of CHD9 in the liver after burn. Furthermore, the U-4-11.1 putative MuERV, which was derived from the U-4-11 U3 probe (isolated from the lung of burn mice), was integrated near a cluster of immunoglobulin gamma (IgG) Fc receptor genes. Among these IgG Fc receptors, Fcgr3a has been described as a susceptibility factor for autoimmune diseases such as systemic lupus erythematosus and rheumatoid arthritis [47-49]. An investigation into the role of the U-4-11.1 putative MuERV in the transcriptional control of IgG Fc receptor genes after burn, especially the Fcgr3a gene, will allow us to better understand interactions between this MuERV locus and nearby IgG Fc receptors.

## Conclusion

In this study, we demonstrated that burn-elicited stress signals were responsible for a differential genome-wide alteration in MuERV expression in a tissue- and U3 promoter-specific manner. Biological properties of the 59 putative MuERVs, which were isolated using the U3 sequences as a probe, were examined *in silico *and their potential roles in post-burn pathologic changes were discussed. Further characterization of the full-length as well as defective MuERVs identified in this study is warranted to gain insights into their biological roles, including their interaction/relationship with neighboring genes, in both normal physiology and disease states of the host.

## Methods

### Animal experiments

Female C57BL/6J mice (Jackson Laboratories, Bar Harbor, ME) were housed according to the guidelines of the National Institutes of Health. The Animal Use and Care Administrative Advisory Committee of the University of California, Davis, approved the experimental protocol. The burn protocol employed in this study has been described previously [20]. Briefly, under general anesthesia, an approximately 18 % total body surface area (TBSA) full-thickness flame burn injury was generated on the shaved back of mice accompanied by immediate resuscitation. Control mice were shaved, anesthetized, and resuscitated, but not burned. Three mice from each group were sacrificed by CO_2 _inhalation for tissue (liver, lung, and kidney) collection at 3 hours and 24 hours after burn. In addition, no treatment control mice (three) were sacrificed by cervical dislocation for tissue collection.

### RT-PCR analysis of MuERV expression

Total RNA isolation and cDNA synthesis were performed based on protocols described previously [20]. Briefly, total RNA was extracted using an RNeasy kit (Qiagen Inc., Valencia, CA) and 100 ng of total RNA from each tissue sample was subjected to reverse transcription using Sensiscript reverse transcriptase (Qiagen Inc.). A set of primers, ERV-U1 (5'-CGG GCG ACT CAG TCT ATC GG-3') and ERV-U2 (5'-CAG TAT CAC CAA CTC AAA TC-3'), were used to amplify the MuERV U3 region. These primers have previously been used to amplify non-ecotropic MuERV U3 regions [50].

### Western blot analysis

Protein extracts were prepared from snap-frozen tissues and Western blot analysis was performed as described previously [51]. Briefly, the membrane, blocked in 5% horse serum, was incubated with goat antibody specific for gp69/71 of Rauscher murine leukemia virus (MLV) (ViroMed Biosafety Laboratories, Camden, NJ) followed by anti-goat-HRP antibody (Jackson ImmunoResearch Laboratories, West Grove, PA). The reactive signal was visualized via chemiluminescence.

### Cloning and sequence analysis

PCR products were purified using a QIAquick PCR Purification kit (Qiagen Inc.) and cloned into the pGEM-T Easy vector (Promega, Madison, WI). Plasmid DNAs for sequencing analysis were prepared using a Qiaprep Spin Miniprep kit (Qiagen Inc.). Sequencing was performed at Davis Sequencing Inc. (Davis, CA) using the ABI Prism 377 DNA sequencer from PE Biosystems (Foster City, CA).

### Multiple alignment and phylogenetic tree analyses

The U3 promoter sequences and conserved 178 amino acid residues of RT sequences were aligned using Lasergene (DNASTAR, Madison, WI) and/or Vector NTI (Invitrogen, Carlsbad, CA) [25]. Phylogenetic trees were established using the neighbor-joining method within the MEGA3 program [52,53]. In order to evaluate the statistical confidence of branching patterns, bootstrapping was performed with 100 replications within the program.

### Transcription regulatory elements on MuERV U3 sequences

The profile of transcription regulatory elements on the U3 promoter sequences was determined using the MatInspector program from Genomatix (Munich, Germany). The parameters were set at a core similarity of 90 %, resulting in a 10 % or less mismatch within the core sequence and the matrix similarity was optimized to reduce false positives [54].

### *In silico *cloning/mapping of putative MuERVs using U3 sequences as a probe and ORF analysis

Putative MuERV sequences were identified by surveying the entire mouse (C57BL/6J) genome database from the NCBI using individual U3 promoter sequences as a probe. Initially, the genomic U3 sequences with greater than 97 % homology with respective U3 probes were selected for further cloning/mapping analyses. We then searched for putative MuERV sequences in the genome ranging from approximately 5 kb to 9 kb and flanked by almost identical LTRs at both 5' and 3' ends. Subsequently, these putative MuERV sequences were subjected to ORF analyses for *gag*, *pol*, and *env *polypeptides using reference retroviral sequences encoding intact polypeptides (GenBank accession number: AF033811, J02255, DQ241301, S80082, M17327, and AAO37285).

### Analyses of tropism, primer binding sites (PBSs), and neighboring cellular genes of putative MuERVs

Cellular tropism of 16 putative full-length MuERVs were determined by *in silico *RFLP analysis using three restriction enzymes, *Bam*HI, *Eco*RI, and *Hin*dIII, using Vector NTI (Invitrogen). The RFLP data of each putative MuERV were compared to the reference profiles for each tropism (ecotropic, xenotropic, polytropic, and modified polytropic) [27]. A stretch of 18 bp, located immediately downstream of the 5' U5 region, was examined to determine PBSs for all putative MuERVs identified in this study. The conserved PBS sequences for tRNA^Proline(P) ^and tRNA^Glutamine(Q) ^were used as a references [55,56]. In addition, host genes residing near (within 110 kb upstream and 110 kb downstream) the individual integration sites of putative MuERVs were mapped based on the NCBI and Ensemble mouse genome database [57].

## Authors' contributions

This study was conceived, designed, and coordinated by KC. DGG participated in its coordination and active scientific discussion. AC and LF performed *in silico *data analyses and participated in the generation of figures, while YKL collected and analyzed the results, finalized the figures, and drafted the manuscript. RT performed the Western blot experiment. All authors read and approved the final manuscript.

## Supplementary Material

Additional file 1 **Figure S1**: Multiple alignment of MuERV U3 sequences related to burn and/or no burn. The 31 unique MuERV U3 sequences isolated from tissues (liver, lung, and kidney) of burn and/or no burn mice were subjected to multiple alignment analysis. Yellow regions have a 100 % homology, white regions are non-similar sequences, blue regions indicate conserved sequences, and dashes represent absence of sequences. The locations of the direct repeat, unique region and TATA box are identified in dotted boxes. An insertion of 190 bp is also indicated in the middle of the alignment. **Table S1**: Profile of transcription regulatory elements of 31 MuERV U3 sequences. A total of 73 transcription regulatory elements were analyzed. Numbers in the box indicate frequency of each transcription regulatory element. Gray box indicates no occurrence of specific transcription regulatory elements. **Table S2**: Neighboring host genes within 110 kb upstream and downstream of integration sites of individual putative MuERVs. A total of 145 neighboring host genes were identified within the search range. Click here for file
