# Supplementary material for: Genome-wide changes in expression profile of murine endogenous retroviruses (MuERVs) in distant organs after burn injury
Source: BMC Genomics. 2007 Nov 28;8:440. doi: 10.1186/1471-2164-8-440 (PMC2241634; doi:10.1186/1471-2164-8-440)
Supplement: Additional file 1 — Figure S1: Multiple alignment of MuERV U3 sequences related to burn and/or no burn. The 31 unique MuERV U3 sequences isolated from tissues (liver, lung, and kidney) of burn and/or no burn mice were subjected to multiple alignment analysis. Yellow regions have a 100 % homology, white regions are non-similar sequences, blue regions indicate conserved sequences, and dashes represent absence of sequences. The locations of the direct repeat, unique region and TATA box are identified in dotted boxes. An insertion of 190 bp is also indicated in the middle of the alignment. Table S1: Profile of transcription regulatory elements of 31 MuERV U3 sequences. A total of 73 transcription regulatory elements were analyzed. Numbers in the box indicate frequency of each transcription regulatory element. Gray box indicates no occurrence of specific transcription regulatory elements. Table S2: Neighboring host genes within 110 kb upstream and downstream of integration sites of individual putative MuERVs. A total of 145 neighboring host genes were identified within the search range. [file 1471-2164-8-440-S1.pdf]

Figure S1

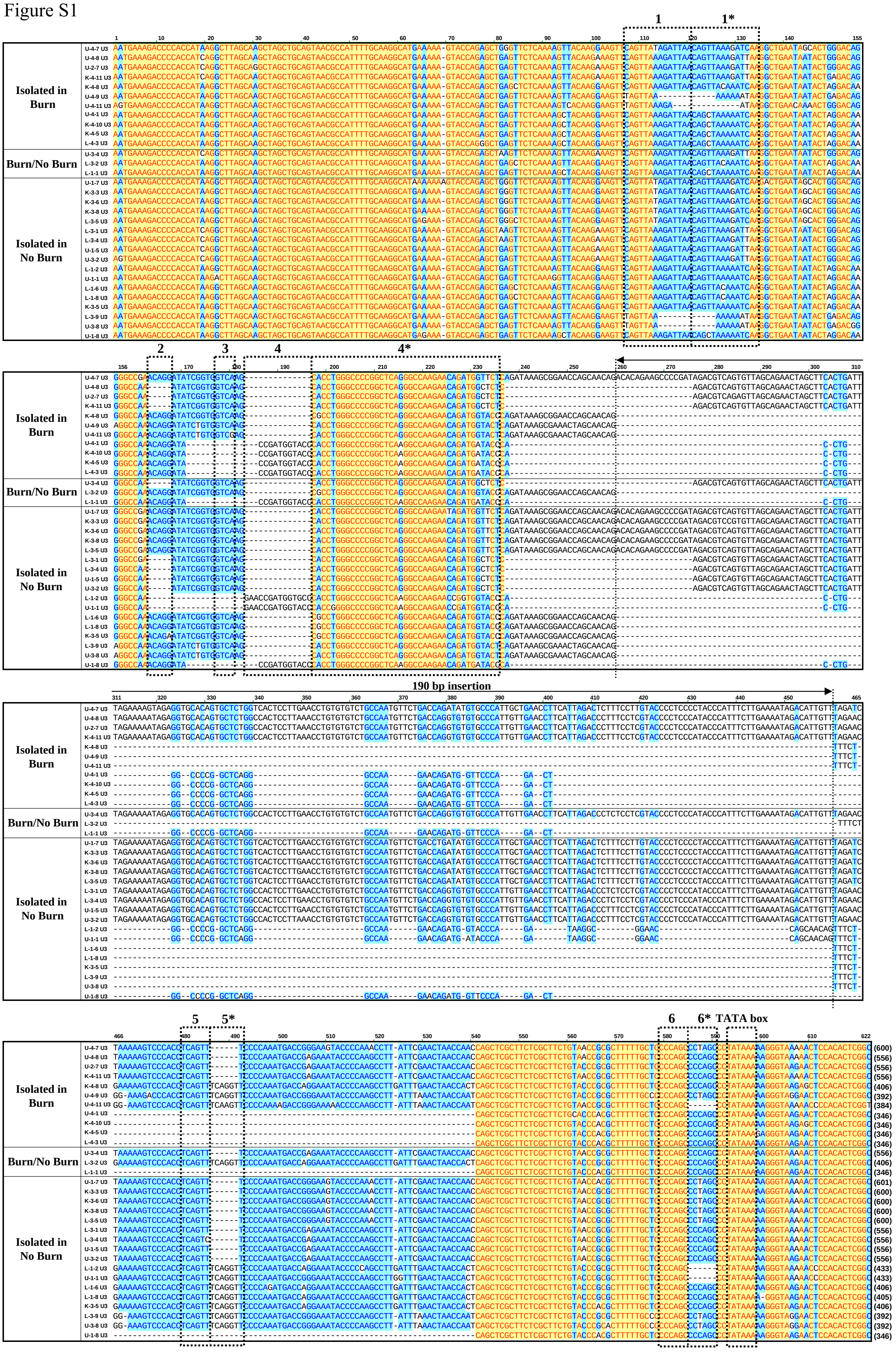
1
1*
2
3
4
4*
5
5*
6
6*
TATA box
190 bp insertion
Isolated in Burn
Burn/No Burn
Isolated in No Burn

Table S1. Profile of transcription regulatory elements on 31 unique MuERV U3 sequences

Group	U3 Clones (size) \ Transcription regulatory elements	AML1/CBFA2 Runt domain binding site	AREB6 (Atp1a1 regulatory element binding factor 6)	Barbiturate-inducible element	B-cell-specific activator protein	Bipartite binding site of VDR/RXR heterodimers [b]	CCAAT/enhancer binding protein	CCAAT/enhancer binding protein beta [a]	Cell cycle-dependent element (CDE)	Cell cycle gene homology region (CHR) [b]	Cellular and viral TATA box elements	c-Ets-2 binding site	c-Myb	DNA binding complex of Lmo2 [b]	Downstream regulatory element-antagonist modulator	E2F	E4BP4 transcriptional repressor	Ecotropic viral integration site 1 encoded factor	Elk-1	Estrogen related receptor	Fork head homologous X (FHXA and FHXB) [b]	Fork head related activator-2 (FOXF2)	GATA binding factor	GATA-binding factor 1 [b]	GATA-binding factor 3	Growth factor independence 1 zinc finger protein Gfi-1B
Isolated in Burn	U-4-7 (600 bp)		2		1		1	1	1		1	1	1		1	1	1	2	1			1	1	1	1	
	K-4-11 (556 bp)		2	1			1		1		1				1	2	1					1				
	U-2-7 (556 bp)		2	1			1		1		1					2										
	U-4-8 (556 bp)		2	1			1		1		1				1	2	1					1				
	K-4-8 (406 bp)				1		1		1	1	1	2				2							1			1
	U-4-9 (392 bp)	1	1				1		1		1	1				1							1			
	U-4-11 (384 bp)	1	1	1			1		1		1	1				2	1					1	1			
	K-4-5 (346 bp)						1				1					2		1	1	1					1	1
	K-4-10 (346 bp)						1				1					2		1	1	1					1	1
	L-4-3 (346 bp)						1				1					2		1	1	1					1	1
	U-4-1 (346 bp)						1				1					2		1	1	1					1	1
Burn/ No Burn	U-3-4 (556 bp)		2	1			1		1		1				1	2	1					1				
	L-3-2 (406 bp)				1		1		1	1	1	2				2							1			1
	L-1-1 (346 bp)						1				1					2		1	1	1					1	1
Isolated in No Burn	U-1-7 (601 bp)		2		1		1				1	1	1		1		1	1	1			1	2		1	
	K-3-3 (600 bp)		2		1		1		1		1	1	1			1	1	2	1			1	1	1	1	
	K-3-6 (600 bp)		2		1		1		1		1	1	1		1	1	1	2	1			1	1	1	1	
	K-3-8 (600 bp)		2		1		1		1		1	1	1		1	1	1	2	1			1	1	1	1	
	L-3-5 (600 bp)		2		1		1		1		1	1	1		1		1	2	1			1	1	1	1	
	L-3-1 (556 bp)		2	1			1		1		1				1	1	1					1				
	L-3-4 (556 bp)		2	1			1		1		1				1	2	1					1				
	U-1-5 (556 bp)		2	1			1		1		1				1	2	1					1				
	U-3-2 (556 bp)		2	1			1		1		1				1	2	1									
	L-1-2 (433 bp)						1		1	1	1	1		1		1				1		1				1
	U-1-1 (433 bp)					1	1		1	1	1			1		1		1		1	1	1			1	1
	L-1-6 (406 bp)				1		1		1	1	1	2				2							1			1
	L-1-8 (405 bp)				1		1		1	1		2				2							1			1
	K-3-5 (404 bp)				1		1		1	1	1	2				2							1			1
	L-3-9 (392 bp)	1	1				1		1		1	1				1							1			
	U-3-8 (392 bp)	1	1				1				1	1				1							1			
	U-1-8 (346 bp)						1				1					1		1	1	1					1	1

Group	U3 Clones (size) \ Transcription regulatory elements	Gut-enriched Krueppel-like factor [b]	Heat shock factor 1	Heat shock factor 2	Hepatic nuclear factor 1	HMGAs (HMGA1, HMGA2) [a]	Hox-1.3, vertebrate homeobox protein	Hypoxia induced factor-1 (HIF-1)	Interferon regulatory factor (IRF)-related protein	Kruppel-like zinc finger protein 219	LBP-1c (leader-binding protein-1c) [b]	LYL1-E12 heterodimeric complex [b]	Mammalian C-type LTR TATA box [b]	Member of the RSRF protein family	MOK-2 protein	Muscle TATA box	Myocyte-specific enhancer factor 2	MyT1-like, C2HC zinc finger factor 1	MyT1 zinc finger transcription factor	Neuron-restrictive silencer factor [b]	NF-kappaB [b]	Nuclear factor 1	Nuclear factor Y (Y-box binding factor)	Nuclear matrix protein 4 (NMP4)	Nuclear Receptor (Nr2e3)	Nuclear respiratory factor 1 (NRF1)
Isolated in Burn	U-4-7 (600 bp)				1									1	1				2	1	1	1	1	1	1	
	K-4-11 (556 bp)		1	1	2		1	1						1	1		2	1	1			1	1	1	1	
	U-2-7 (556 bp)		1	1	2		1	1						1	1		2	1	1			2	1	1	1	
	U-4-8 (556 bp)		1	1	2		1	1						1	1		2	1	1			2	1	1	1	
	K-4-8 (406 bp)				1									1		1			2			1	1	2	1	1
	U-4-9 (392 bp)		1				1		1	1				1			1		2		1	1	2	1	1	
	U-4-11 (384 bp)		1						1					1					1			2	2	2	1	
	K-4-5 (346 bp)				1	1													1			2	1	1		
	K-4-10 (346 bp)				1	1								1					1			2	1	1		
	L-4-3 (346 bp)				1	1								1					1			2	1	1		
	U-4-1 (346 bp)				1	1								1					1			2	1	1		
Burn/ No Burn	U-3-4 (556 bp)		1	1	2		1	1						1			2	1	1			2	1	1	1	
	L-3-2 (406 bp)				1									1		1			2			1	1	2	1	1
	L-1-1 (346 bp)				1	1								1					1			2	1	1		
Isolated in No Burn	U-1-7 (601 bp)				1						1	1	1	1	1		1		2	1	1	1	1		1	
	K-3-3 (600 bp)				1									1	1		1		2	1	1	1	1	1	1	
	K-3-6 (600 bp)				1									1	1		1		2	1	1	1	1	1	1	
	K-3-8 (600 bp)				1									1	1		1		2	1	1	1	1	1	1	
	L-3-5 (600 bp)				1									1	1		1		2	1	1	1	1		1	
	L-3-1 (556 bp)		1	1	2		1	1						1			2	1	1			1	1	1	1	
	L-3-4 (556 bp)		1	1	2		1	1						1			2	1	1			2	1	1	1	
	U-1-5 (556 bp)		1	1	2		1	1						1	1		2	1	1			2	1	1	1	
	U-3-2 (556 bp)		1	1	2		1	1						1	1		2	1	1			2	1	1	1	
	L-1-2 (433 bp)				1									1					2			2		2	1	
	U-1-1 (433 bp)	1			1									1					1		1	2		2	1	
	L-1-6 (406 bp)				1									1		1			2			1	1	2	1	1
	L-1-8 (405 bp)	1			1								1			1			2			1	1	2	1	1
	K-3-5 (404 bp)				1									1		1			2			1	1	2	1	1
	L-3-9 (392 bp)		1				1		1	1				1			1		2		1	1	2	1	1	
	U-3-8 (392 bp)		1				1		1	1				1			1		2		1	1	2	1	1	
	U-1-8 (346 bp)				1	1								1					1			2	1			

Group	U3 Clones (size) \ Transcription regulatory elements	PAR-type chicken vitellogenin promoter-binding protein	PAX6 paired domain [a]	PBX - HOXA9 binding site	PPAR/RXR heterodimers [b]	Promyelocytic leukemia zink finger [b]	PTF1 binding sites	Ras-responsive element binding protein 1	Retinoic acid receptor DR5 site	SF1 steroidogenic factor 1	Signal transducers and activators of transcription	Spi-B transcription factor (Spi-1/PU.1 related)	Stem cell zinc finger protein (SZF1) [a]	SWI/SNF related elements [b]	Tal-1beta/HEB heterodimer	Tal-1beta/ITF-2 heterodimer	TCF11/MafG heterodimers	Thing1/E47 heterodimer [a]	Transcriptional repressor (ZNF202)	v-Myb, AMV v-myb	Winged helix protein	Yin and Yang 1 repressor sites	PAX2 paired domain protein	Zinc finger transcription factor RU49
Isolated in Burn	U-4-7 (600 bp)	1		1	1	1	1	1	1			1			1					1		1		2
	K-4-11 (556 bp)	1		1			1	1	1							1	1		1	1		1	1	1
	U-2-7 (556 bp)	1		1			1	1	1							1	1		1	1		1	1	1
	U-4-8 (556 bp)	1		1			1	1	1							1	1		1	1		1	1	1
	K-4-8 (406 bp)							1	1			1	1		1				1	1		1		1
	U-4-9 (392 bp)							1			1				1				1	1		1		1
	U-4-11 (384 bp)	1						1			1				1					1		1		1
	K-4-5 (346 bp)								1	1					2			1	1	1	1	1		1
	K-4-10 (346 bp)								1	1			1		2			1	1	1	1	1		1
	L-4-3 (346 bp)		1						1	1					2			1	1	1	1	1		1
	U-4-1 (346 bp)								1	1					2			1	1	1	1	1		1
Burn/ No Burn	U-3-4 (556 bp)	1		1			1	1	1							1	1		1	1		1		1
	L-3-2 (406 bp)							1	1			1			1				1	1		1		1
	L-1-1 (346 bp)								1	1					2			1	1	1	1	1		1
Isolated in No Burn	U-1-7 (601 bp)	1		1	1	1	1	1	1			1								1	1	1		2
	K-3-3 (600 bp)			1	1	1	1	1	1			1			1					1		1		2
	K-3-6 (600 bp)	1		1	1	1	1	1	1			1			1					1		1		2
	K-3-8 (600 bp)	1		1	1	1	1	1	1			1			1					1		1		2
	L-3-5 (600 bp)	1		1	1	1	1	1	1			1			1					1		1		2
	L-3-1 (556 bp)	1		1			1	1	1							1	1		1	1		1		1
	L-3-4 (556 bp)	1		1			1	1	1							1	1		1	1		1		1
	U-1-5 (556 bp)	1		1			1	1	1							1	1		1	1		1		1
	U-3-2 (556 bp)	1		1			1	1	1							1	1		1	1		1	1	1
	L-1-2 (433 bp)							1	1	1					1					1		1		1
	U-1-1 (433 bp)				1			1	1	1					1					1		1		1
	L-1-6 (406 bp)								1			1			1				1	1		1		1
	L-1-8 (405 bp)							1	1			1			1				1	1		1		1
	K-3-5 (404 bp)							1	1			1			1				1	1		1		1
	L-3-9 (392 bp)							1			1				1				1	1		1		1
	U-3-8 (392 bp)							1			1			1	1				1	1		1		1
	U-1-8 (346 bp)								1	1					2			1	1	1	1	1		1

A total of 73 transcription regulatory elements were analyzed. Numbers in the box indicate frequency of each transcription regulatory element. Gray box indicates no occurrence of specific transcription regulatory elements.
[a] predominantly isolated in “Isolated in Burn” group transcription regulatory element. [b] predominantly isolated in “ Isolated in No Burn” group transcription regulatory element.

Table S2. Neighboring host genes within 110 kb upstream and downstream of integration sites of individual putative MuERVs

Ch*	Virus	Gene	Or**	Description
1	**K4-11.1a**	Selp	(+/+)	selectin, platelet
		F5	(+/+)	coagulation factor V
		S1C19a2	(+/+)	solute carrier family 19 (thiamine transporter), member 2
		Nme7	(+/+)	non-metastatic cells 7
		Blzf1	(+/-)	basic leucine zipper nuclear factor 1
	**K-4-11.1b**[c]	Nek2	(+/+)	never in mitosis gene a -related expressed kinase 2
		Slc30a1	(+/+)	solute carrier family 30 (zinc transporter), member 1
	**U-4-8.1**[c]	Degs1	(+/-)	degenerative spermatocyte homolog 1
		Fbxo28	(+/-)	F-box protein 28
	**U-4-11.1**[c]	Dusp12	(+/+)	dual specificity phosphatase12
		Fcgr3a	(+/+)	Fc receptor, IgG, low affinity IIIa
		Fcgr2b, 3	(+/-)	Fc receptor, IgG, low affinity IIb, III
		Fcrl a, b	(+/-)	Fc receptor like a, b
		Atf6	(+/-)	activating transcription factor 6
2	**K-4-11.2**	Nr4a2	(-/-)	nuclear receptor subfamily 4, group A, member 2
		Gpd2	(-/+)	glycerol phosphate dehydrogenase 2, mitochondrial
3	**K-3-3.3b**	Fubp1	(+/+)	far upstream element (FUSE) binding protein 1
		Usp33	(+/+)	ubiquitin specific peptidase 33
		Zzz3	(+/+)	zinc finger, ZZ domain containing 3
		Nexn	(+/-)	nexilin
4	**L-1-1.4**[a]	Fgr	(+/+)	gardner-rasheed feline sarcoma viral oncogene homolog
		Ahdc1	(+/+)	AT hook, DNA binding motif, containing 1
		Wasf2	(+/+)	WAS protein family, member 2
	**K-3-6.4**[b]	Efcbp1	(-/-)	EF hand calcium binding protein 1
		Tmem64	(-/+)	transmembrane protein 64
	**K-3-3.4**	Ankrd6	(-/-)	ankyrin repeat domain 6
	**K-4-11.4a**	Lepr	(-/+)	leptine receptor
	**K-4-11.4b**	Scp2	(-/-)	sterol carrier protein 2, liver
		Echdc2	(-/+)	enoyl coenzyme A hydratase domain containing 2
5	**K-3-3.5b**	Cpeb2	(+/+)	cytoplasmic polyadenylation element binding protein 2
	**K-3-3.5a**	Myl2	(+/+)	myosin, light polypeptide 2
		Ppp1cc	(+/+)	protein phosphatase 1, catalytic subunit, gamma isoform
		Hvcn1	(+/+)	hydrogen voltage-gated channel 1
		Pptc7	(+/+)	PTC7 protein phophatase homolg (S. cerevisiae)
		Ccdc63	(+/-)	coiled-coil domain containing 63
	**U-1-5.5a**[b]	Prom1	(+/-)	prominin 1
	**U-1-5.5b**[b]	Srp72	(+/+)	signal recognition particle 72
		Arl9	(+/+)	ADP-ribosylation factor-like 9
		Paics	(+/+)	phosphoribosylaminoimidazole carboxylase
		Ppat	(+/-)	phosphoribosyl pyrophosphate amidotransferase
	**U-4-8.5**[c]	Trrap	(+/-)	transformation/transcription domain-associated protein
		Tmem130	(+/-)	transmembrane protein 130
	**L-3-9.5**[b]	Srpk2	(-/-)	serine/arginine-rich protein specific kinase 2
		Pus7	(-/-)	psedouridylate synthase 7 homolg (S. cerevisiae)
		Rint1	(-/+)	RAD50 interactor 1
6	**K-3-6.6**[b]	Suclg1	(-/+)	succinate-CoA ligase, GDP-forming, alpha subunit
7	**K-3-6.7**[b]	Trpm1	(+/+)	transient receptor potential cation channel, subfamily M
	**K-4-11.7a**	Usp29	(-/+)	ubiquitin specific peptidase 29
		Zfp418	(-/+)	zinc finger protein 418
	**K-4-11.7c**	Atp4a	(+/+)	ATPase, H+/K+ exchanging, gastric, alpha polypeptide
		Krtdap	(+/+)	keratinocyte differentiation associated protein
		Sbsn	(+/+)	suprabasin
		Wbp7	(+/-)	WW domain binding protein 7
		Zbtb32	(+/-)	zinc finger and BTB domain containing 32
		Upk1a	(+/-)	uroplakin 1A
		Cox6b1	(+/-)	cytochrome c oxidase, subunit VIb polypeptide 1
		Etv2	(+/-)	ets variant gene 2
		Tmem147	(+/-)	transmembrane protein 147
		Gapdhs	(+/-)	glyceraldehyde-3-phosphate dehydrogenase
	**K-4-11.7d**	Nucb2	(+/+)	nucleobindin 2
		Pik3c2a	(+/-)	phosphatidylinositol 3-kinase, C2 domain, α polypeptide
8	**K-3-6.8**[b]	Rpl13	(+/+)	ribosomal protein L13
		Dpep1	(+/+)	dipeptidase 1 (renal)
		Cdk10	(+/+)	cyclin-dependent kinase (CDC2-like) 10
		Zfp276	(+/+)	zinc finger protein (C2H2 type) 276
		Spg7	(+/+)	spastic paraplegia 7 homolg (human)
		Fanca	(+/-)	fanconi anemia, complementation group A
		Sult5a1	(+/-)	sulfotransferase family 5A, member 1
		Pcoln3	(+/-)	procollagen (type III) N-endopeptidase
	**U-3-4.8**[a]	Cotl1	(-/-)	coactosin-like 1 (Dictyostelium)
		Usp10	(-/+)	ubiquitin specific peptidase 10
		Crispld2	(-/+)	cysteine-rich secretory protein LCCL domain containing 2
	**L-1-2.8a**	Zfp42	(-/-)	zinc finger protein 42
	**L-1-2.8b**	Asna1	(-/-)	arsA arsenite transporter, ATP-binding, homolog 1
		Tnpo2	(-/+)	transportin 2 (import 3, karyopherin beta 2b)
		Fbxw9	(-/+)	F-box and WD-40 domain protein 9
		Dhps	(-/+)	deoxyhypusine synthase
		Man2b1	(-/+)	mannosidase 2, alpha B1
		Olfr371	(-/+)	olfactory receptor 371
9	**L-1-2.9**	Sorl1	(-/-)	sortilin-related receptor, LDLR class A repeats-containing
10	**K-3-3.10a**	Vip	(-/-)	vasoactive intestinal polypeptide
		Rgs 17	(-/+)	regulator of G-protein signaling 17
		Fbxo5	(-/+)	F-box protein 5
		Mtrf1l	(-/+)	mitochondrial translational release factor 1-like
	**K-3-3.10b**	Slc2a12	(+/+)	solute carrier family 2, member 12
		Tbpl1	(+/-)	TATA box binding protein-like 1
		Tcf21	(+/-)	transcription factor 21
	**K-4-11.10a**	Ust	(-/-)	uronyl-2-sulfotransferase
	**K-4-11.10b**	Zbtb24	(+/+)	zinc finger and BTB domain containing 24
		Mical1	(+/+)	microtubule associated monoxygenase
		Cd164	(+/+)	CD164 antigen
		Smpd2	(+/+)	sphingomyelin phosphodiesterase 2
	**K-3-3.11a**	Drg2	(-/+)	developmentally regulated GTP binding protein 2
		Myo15	(-/+)	myosin XV
		Alkbh5	(-/+)	alkB, alkylation repair homolog 5 (E.coli)
	**K-3-3.11c**	Dhx40	(+/-)	DEAH (Asp-Glu-Ala-His) box polypeptide 40
		Ypel2	(+/-)	Yippee-like 2 (Drosophila)
	**K-4-11.11b**	Akap1	(-/-)	A kinase (PRKA) anchor protein 1
		Scpep1	(-/-)	serine carboxypeptidase 1
		Coil	(-/+)	coilin
		Trim25	(-/+)	tripartite motif protein 25
	**K-3-6.12**[b]	Ctse	(+/+)	cathepsin E
		Sos2	(+/-)	son of sevenless homolog 2 (Drosophila)
13	**K-3-3.13**	Hist1h2ai	(+/+)	histone 1, H2ai
		Hist1h2ab	(+/+)	histone 1, H2ab
		Hist1h2bb	(+/+)	histone 1, H2bb
		Hist1h2bc	(+/+)	histone 1, H2bc
		Hist1h2bf	(+/+)	histone 1, H2bf
		Hist1h2bm	(+/+)	histone 1, H2bm
		Hist1h3g	(+/+)	histone 1, H3g
		Hist1h4a	(+/+)	histone 1, H4a
		Olfr1359	(+/+)	olfactory receptor 1359
	**K-3-5.13**	Zfp712	(-/+)	zinc finger protein 712
	**K-4-11.13**	Tnpo1	(-/-)	transportin 1
		Rpl27a	(-/+)	ribosomal protein L27a
14	**L-1-2.14**	Mrpl52	(+/+)	mitochondrial ribosomal protein L52
		Mmp14	(+/+)	matrix metallopeptidase 14 (membrane-inserted)
		Lrp10	(+/+)	low-density lipoprotein receptor-related protein 10
		Rem2	(+/+)	rad and gem related GTP binding protein 2
		Cdh24	(+/+)	cadherin-like 24
		Prmt5	(+/-)	protein arginine N-methyltransferase 5
		Jub	(+/-)	ajuba
		Psmb5	(+/-)	proteasome subunit beta type 5 precursor
		Acin1	(+/-)	apoptotic chromatin condensation inducer 1
15	**U-1-5.15**[b]	Bop1	(-/-)	block of proliferation 1
		Nfkbil2	(-/-)	nuclear factor of k light peptide gene enhancer in B cells inhibitor-like 2
		Slc39a4	(-/-)	solute carrier family 39 (zinc transporter), member 4
		Dgat1	(-/-)	diacylglycerol O-acyltransferase 1
		Scrt1	(-/-)	scratch homolog 1, zinc finger protein (Drosophila)
		Fbxl6	(-/-)	F-box and leucine-rich repeat protein 6
		Cpsf1	(-/-)	cleavage and polyadenylation specific factor 1
		Vps28	(-/-)	vacuolar protein sorting 28 (yeast)
		Foxih1	(-/-)	forkhead box H1
		Cyhr1	(-/-)	cysteine and histidine rich -1
		Scx	(-/+)	scleraxis
		Hsf1	(-/+)	heat shock factor 1
		GPR172b	(-/+)	G protein-coupled receptor 172B
		Adck5	(-/+)	aarF domain containing kinase 5
		Ppp1r16a	(-/+)	protein phosphatase 1, regulatory (inhibitor)
18	**U-4-8.18**[c]	Mbp	(+/+)	myelin basic protein
		Zfp236	(+/-)	zinc finger protein 236
19	**K-4-11.19**	Lgi1	(+/+)	leucine-rich repeat LGI family, member 1
		Tmem20	(+/+)	transmembrane protein 20

Putative MuERVs isolated with 100% homology with respective U3 probes: [a] Isolated in Burn/No Burn, [b] Isolated in No Burn, and [c] Isolated in Burn. *chromosome
** orientation (virus/gene)
